# Identification and profiling of microRNAs during gonadal development in the giant freshwater prawn *Macrobrachium rosenbergii*

**DOI:** 10.1038/s41598-019-38648-x

**Published:** 2019-02-20

**Authors:** Xue Liu, Bi-Yun Luo, Jian-Bin Feng, Ling-Xia Zhou, Ke-Yi Ma, Gao-Feng Qiu

**Affiliations:** 1 National Demonstration Center for Experimental Fisheries Science Education (Shanghai Ocean University), Shanghai, China; 2 Key Laboratory of Exploration and Utilization of Aquatic Genetic Resources (Shanghai Ocean University), Ministry of Education, Shanghai, China; 3 Key Laboratory of Freshwater Aquatic Genetic Resources, Ministry of Agriculture (Shanghai Ocean University), Shanghai, China; 4 Shanghai Engineering Research Center of Aquaculture (Shanghai Ocean University), Shanghai, China

## Abstract

As post-transcriptional regulators, microRNAs (miRNAs) play an important role in growth and reproductive processes. So far, there is limited information regarding crustacean miRNAs. To explore the potential role of miRNAs in the gonadal development of the prawn *Macrobrachium rosenbergii*, we constructed seven small RNA libraries from ovarian and testicular tissues at various stages using somatic tissue as the control. A total of 1,954 known and 129 novel miRNAs were retrieved. By comparing differentially expressed miRNAs (DEMs) between testes and ovaries, forty-one miRNAs were identified with sex-biased expression patterns, including 17 ovary-biased and 24 testis-biased patterns. Furthermore, the putative target genes of the sex-biased miRNAs, such as cyclin L1, mitogen-activated protein kinase 7 (MAPK 7), heat shock protein (HSP), and zinc finger protein, were significantly enriched in many reproduction-related pathways including the Gonadotropin-releasing hormone (GnRH) pathway, glycolysis, gluconeogenesis pathway, ovarian steroidogenesis, estrogen signaling pathway, MAPK pathway, Wnt pathway, and insulin signaling pathway, implicating potential regulatory roles of miRNAs in reproduction. These data aid in the further investigation of the mechanism of gonadal development and reproductive regulation mediated by miRNA in *M*. *rosenbergii*.

## Introduction

MicroRNAs (miRNAs) are small single-stranded non-coding RNA molecules. As key post-transcriptional regulators, miRNAs in animals are capable of controlling gene expression mostly through interaction with the 3′ untranslated region (3′UTR) of the target mRNA for degradation or translational inhibition^1^. A large number of protein-coding genes (PCGs) have been revealed to be targeted by miRNAs. It is interesting to note that a single miRNA molecule can target thousands of mRNAs, and that each mRNA could have recognition sequences for multiple miRNAs as well. More than one third of the human genome has been determined to be regulated by miRNAs^2,3^.

miRNAs are involved in the regulation of a variety of life processes^4,5^. Recent evidence has shown that miRNAs play an important role in the development of reproductive systems and the differentiation of germ cells^6–8^. Statistical, genome-wide analysis of miRNAs discovered many differentially expressed miRNAs (DEMs) during gonadal development in mammals, including mouse (*Mus musculus*)^9,10^, pig (*Sus scrofa*)^11^, cattle (*Bos Taurus*)^12^, and sheep (*Ovis aries*)^13^. Sex-biased miRNAs may be directly involved in the differential expression of many target genes, which contribute to different sexual traits during gonadal development^14^. In *Acipenser schrenckii*, 37 miRNAs were detected exclusively in the gonad and 148 miRNAs exhibited sex-biased expression, including 83 female-biased and 65 male-biased miRNAs. Based on these DEMs, twenty-five putative gametogenesis-related target genes were predicted, including anti-Mullerian hormone (*Amh*), winged helix/forkhead transcription factor (*Foxl2*), and *Vasa*^15^. Subsequent studies also revealed many DEMs in sea urchin (*Strongylocentrotus nudus*)^16^, tilapia (*Oreochromis niloticus*)^17^, and common carp (*Cyprinus carpio)*^18^, suggesting that miRNAs involved in gonadal development are mediated by their differential expression of sexual dimorphism.

Gonadal development involves a series of complex regulatory processes at transcriptional and post-transcriptional levels. Although efforts have been made to study this regulation in Decapoda crustaceans, only a few studies have focused on post-transcriptional gene regulation. Furthermore, miRNAs related to gonadal development have only been reported in the Chinese mitten crab (*Eriocheir sinensis*)^8,19^ and swimming crab (*Portunus trituberculatus*)^7^. In *E*. *sinensis*, miR-2 and miR-133 were demonstrated to down regulate cyclin B gene expression during meiotic oocyte maturation^19^. Moreover, some miRNAs (let-7c, miR-21-5p, and miR-17-3p) displayed different expression patterns at various stages of testicular development and may be involved in spermatogenesis^8^. In *P*. *trituberculatus*, 44 ovarian and 31 testicular miRNAs were preferentially expressed^7^. Furthermore, the expression of miR-7 and miR-87 was significantly increased and they were predicted to target cell division cycle 4 (Cdc4) and cyclin-dependent kinase 2 (Cdk2)^7^, both of which are critical for controlling the G1-to-S-phase transition during the cell cycle^20,21^. In the giant freshwater prawn *Macrobrachium rosenbergii*, however, there is limited information currently available regarding miRNAs during gonadal development.

The prawn *M*. *rosenbergii* is a commercially valuable decapod species that is widely cultured in southern and southeastern Asia as well as in the western Pacific^22,23^. During the last decade, there has been significant development in farming technology of *M*. *rosenbergii* in China, with an average annual farming expansion rate of 15.28%^24^. Although this increased productivity and technology has achieved great progress, there have been numerous developmental problems. Furthermore, annual production is not very stable due to the limitation of breeding and farming technology, such as inbreeding and disease^24^. Therefore, it is necessary to focus on the mechanism of gonadal development for breeding and farming technology optimization in *M*. *rosenbergii*.

By taking advantage of high-throughput next-generation sequencing (NGS) technology, we screened miRNAs in from seven different *M*. *rosenbergii* miRNA libraries. During this process we were able to identify novel miRNAs and DEMs for the first time in *M*. *rosenbergii*. We further conducted miRNA expression profile analysis and the functional annotation of DEMs by Gene Ontology (GO) and Kyoto Encyclopedia of Genes and Genomes (KEGG) to explore potential roles of miRNAs in gonadal development. The results from this study provide valuable information regarding the regulatory roles of miRNAs in gonadal development and reproductive control in *M*. *rosenbergii*.

## Results

### Identification of miRNAs from *M*. *rosenbergii*

**Figure 1:**
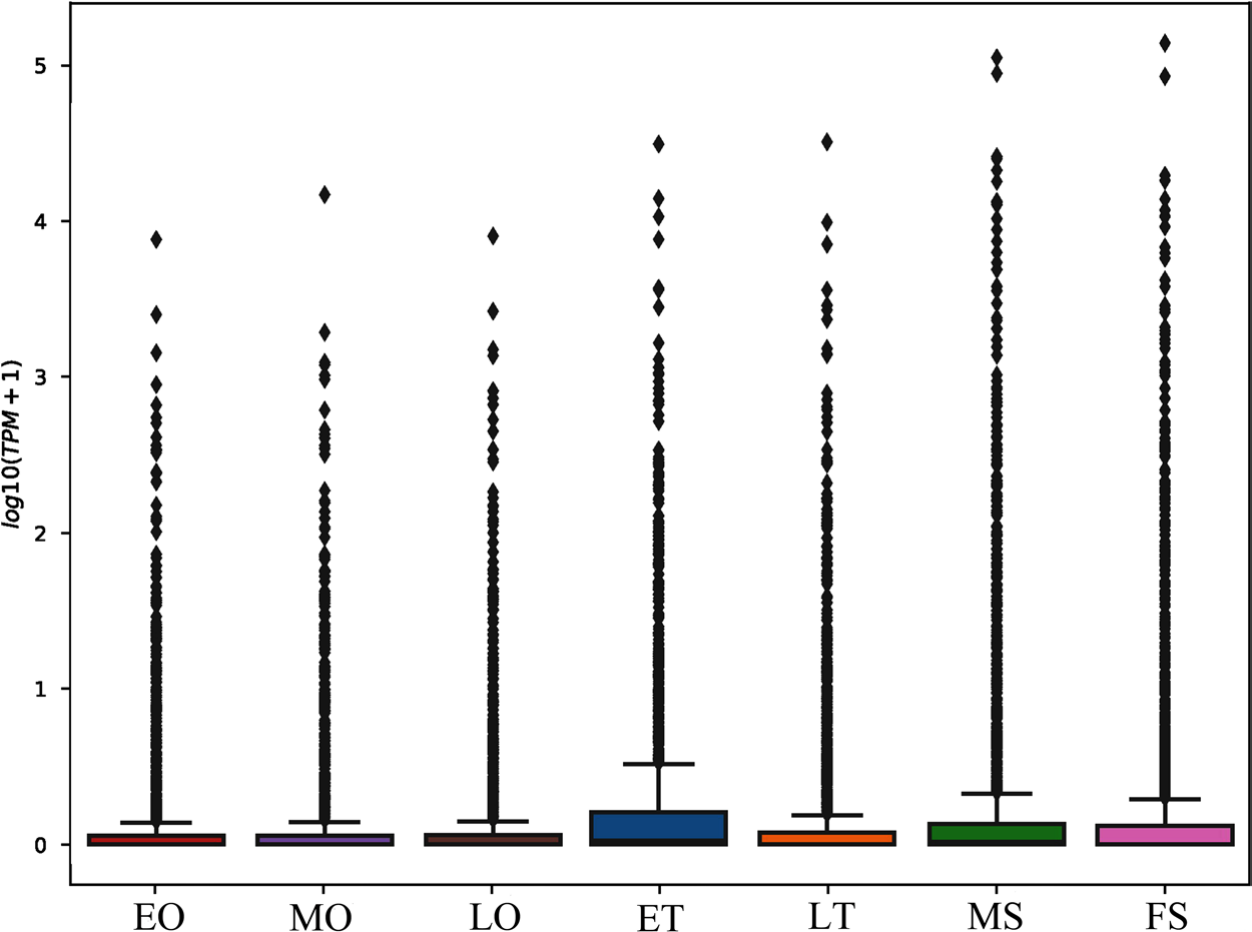
Box-whisker plot of miRNA expression among seven libraries. ET, early-middle testis; LT, late testis; EO, early ovary; MO, middle ovary; LO, late ovary; MS, male somata; and FS, female somata.

We constructed seven cDNA libraries of small RNAs from early-middle (ET) and late (LT) developing testis; early (EO), middle (MO) and late developing (LO) ovaries; and male (MS) and female (FS) somata. A total of 19,316,857; 30,914,458; 51,441,365; 31,871,854; 29,986,842; 29,092,334; and 25,530,458 raw reads were initially generated from the seven libraries, respectively. After filtering out low quality sequences, there were 17,837,427; 26,153,111; 41,516,524; 27,583,544; 26,887,205; 24,940,628; and 22,053,365 clean reads remaining in the corresponding libraries. By comparing the small RNA sequences with those sequences in the Rfam, non-coding RNAs in all of the libraries were categorized into rRNA, snRNA, tRNA, known miRNA and those without annotation (Table 1). As shown in Table 1, most of the small RNAs were unannotated with a range from 10,003,331 to 34,476,353 sequences, implying that small RNA information is still underdeveloped for decapod species. After removing the repeat reads, known and novel miRNAs were retrieved from each library as shown in Table 2. A total of 1,954 known and 129 novel miRNAs were obtained. The highest number of the known miRNAs was found in the ET library, while three ovary libraries generated the highest number of novel miRNAs, displaying 49, 46, and 44 novel miRNAs, respectively. To get a rudimentary understanding for the distribution of the miRNA among seven libraries, we further analyzed the data using box-whisker plot (Fig. 1).

**Table 1:** Distribution of reads in theseven small RNA libraries.

Category	ET	LT	EO	MO	LO	MS	FS
	reads	reads	reads	reads	reads	reads	reads
rRNA	3,769	33,136	48,257	20,316	40,387	2,158	5,080
tRNA	1,171	5,584	22,061	13,703	16,964	1,464	2,728
snRNA	4,733	9,983	18,753	10,105	9,660	3,380	2,728
miRNA	1,742,122	1,965,016	758,290	723,904	558,479	10,377,965	8,546,491
unannotation	13,974,481	20,515,216	34,476,353	24,099,130	22,000,208	11,351,482	10,003,331

Note: ET, early-middle testis; LT, late testis; EO, early ovary; MO, middle ovary; LO, late ovary; MS, male somata; and FS, female somata.

**Table 2:** Numbers of known and novel miRNAs in seven small RNA libraries.

Library	KnownmiRNA	NovelmiRNA
ET	1,148	36
LT	888	38
EO	937	49
MO	854	46
LO	850	44
MS	1,009	33
FS	957	36

Note: ET, early-middle testis; LT, late testis; EO, early ovary; MO, middle ovary; LO, late ovary; MS, male somata; and FS, female somata.

### Length distribution of small RNAs

**Figure 2:**
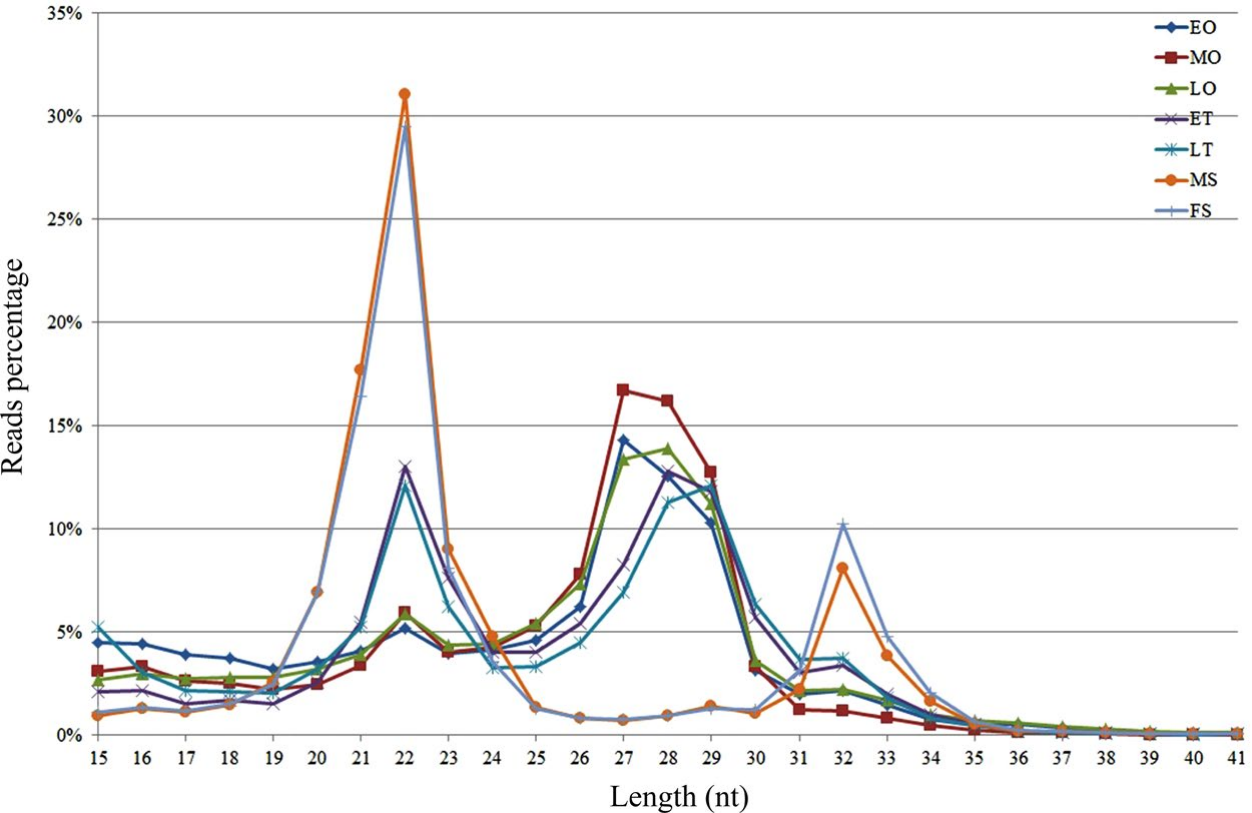
Length distribution of clean reads. The nucleotide length of miRNAs is shown on the X-axis; the percentage of different nucleotide lengths in the sized miRNAs among the total miRNA population is shown on the Y-axis. ET, early-middle testis; LT, late testis; EO, early ovary; MO, middle ovary; LO, late ovary; MS, male somata; and FS, female somata.

The length distribution of the high-quality reads varied through markedly different bimodal patterns in size distribution (Fig. 2). One peak for the 22 nucleotide (nt) size class represents the typical miRNA of Dicer-processed product. Other peaks for 27 nt or 32 nt mostly represent longer piwi-interacting RNAs (piRNAs), which are endogenous small non-coding RNA molecules. As Fig. 2 showed, in EO, MO, and LO, the peak represented by the longest size class (27–29 nt) was much higher than for the peaks of the 21–23 nt size class, indicating that piRNAs were more abundant in *M*. *rosenbergii* ovary. On the other hand, the amount of miRNAs and piRNAs were not significantly different in ET and LT. In comparison with the status in gonad, the length distributions were quite different in MS and FS. MiRNAs were more abundant with a percentage of approximately 30% in MS and FS. Additionally, the length of piRNAs ranged from 31 to 33 nt, which was longer than that those of the gonads.

### Nucleotide bias of miRNAs

The nucleotide bias of miRNAs at each position was analyzed (Supplementary Fig. S1). The results showing that U and A were the dominant nucleotides in all seven libraries. Interestingly, there were relatively fewer U on bases 2–4 and A was generally distributed on the 10^th^ base. This is because the localization of the 10^th^ base is generally the cleavage site of the miRNA when its target gene is sheared. Analysis on the first nucleotide bias of miRNAs showed that U was the predominantly common first nucleotide between the lengths of 15–25 nt, with its proportion exceeding 50% (Supplementary Fig. S2). Nevertheless, A was the dominant first nucleotide of 26–27 nt, and its proportion ranged from 75% to 100%.

### GO and KEGG annotation

**Figure 3:**
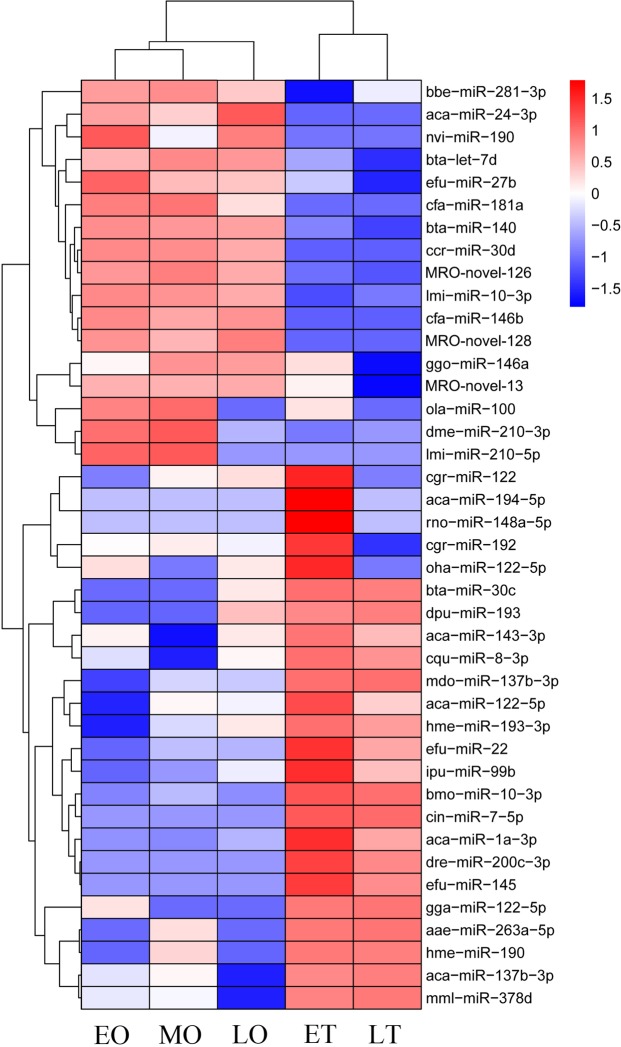
Heat-map of 41 differentially expressed miRNAs between ovary and testis. The heat-map was constructed based on the relative expression level of miRNA (TPM fold change > 2, *P* < 0.05). The color indicates the log2-fold change from high (red) to low (blue), as indicated by the color scale. The name of miRNAs and the libraries to which they belong are shown on the right side. The novel miRNAs have been revised as MRO-novel-**, the others are known miRNAs. ET, early-middle testis; LT, late testis; EO, early ovary; MO, middle ovary; LO, late ovary; MRO, *Macrobrachium rosenbergii*; aae, Aedes aegypti; aca, Anolis carolinensis; bbe, Branchiostoma belcheri; bmo, Bombyx mori; bta, Bos taurus; ccr, Cyprinus carpio; cfa, Canis familiaris; cgr, Cricetulus griseus; cin, Ciona intestinalis; cqu, Culex quinquefasciatus; dme, Drosophila melanogaster; dpu, Daphnia pulex; dre, Danio rerio; efu, Eptesicus fuscus; gga, Gallus gallus; ggo, Gorilla gorilla; hme, Heliconius melpomene; ipu, Ictalurus punctatus; lmi, Locusta migratoria; mdo, Monodelphis domestica; mml, Macaca mulatta; nvi, Nasonia vitripennis. oha, Ophiophagus Hannah; rno, Rattus norvegicus.

We obtained DEMs by comparing the expression of miRNAs between different libraries. By comparing the differentially expressed miRNAs (DEMs) between testes and ovaries, forty-one miRNAs were identified with sex-biased expression patterns, including 17 ovary-biased and 24 testis-biased miRNAs (Fig. 3). For instance, miR-194-5p and miR-148a-5p demonstrated higher expression in the ET library, suggesting they could play important roles in early testicular development. In contrast, miR-24-3p was up-regulated in ovary, especially in LO the library, indicating that miR-24-3p may be involved in ovarian maturation. Subsequently, Gene ontology (GO) annotation was performed to elucidate the function of the target genes identified as being potentially regulated by DEMs. A total of 253 potential target genes were enriched between the ovaries and testes. Amongst the three categories of biological process, cellular component, and molecular function, the most enriched GO terms were “transcription, DNA-templated”, “cytoplasm”, and “poly (A) RNA binding”, respectively (Supplementary Fig. S3A). Additionally, many predicted target genes were involved in gonadal development, such as cyclin L1, mitogen-activated protein kinase 7 (MAPK 7), heat shock protein (HSP), and zinc finger protein (Supplementary Table S1).

**Figure 4:**
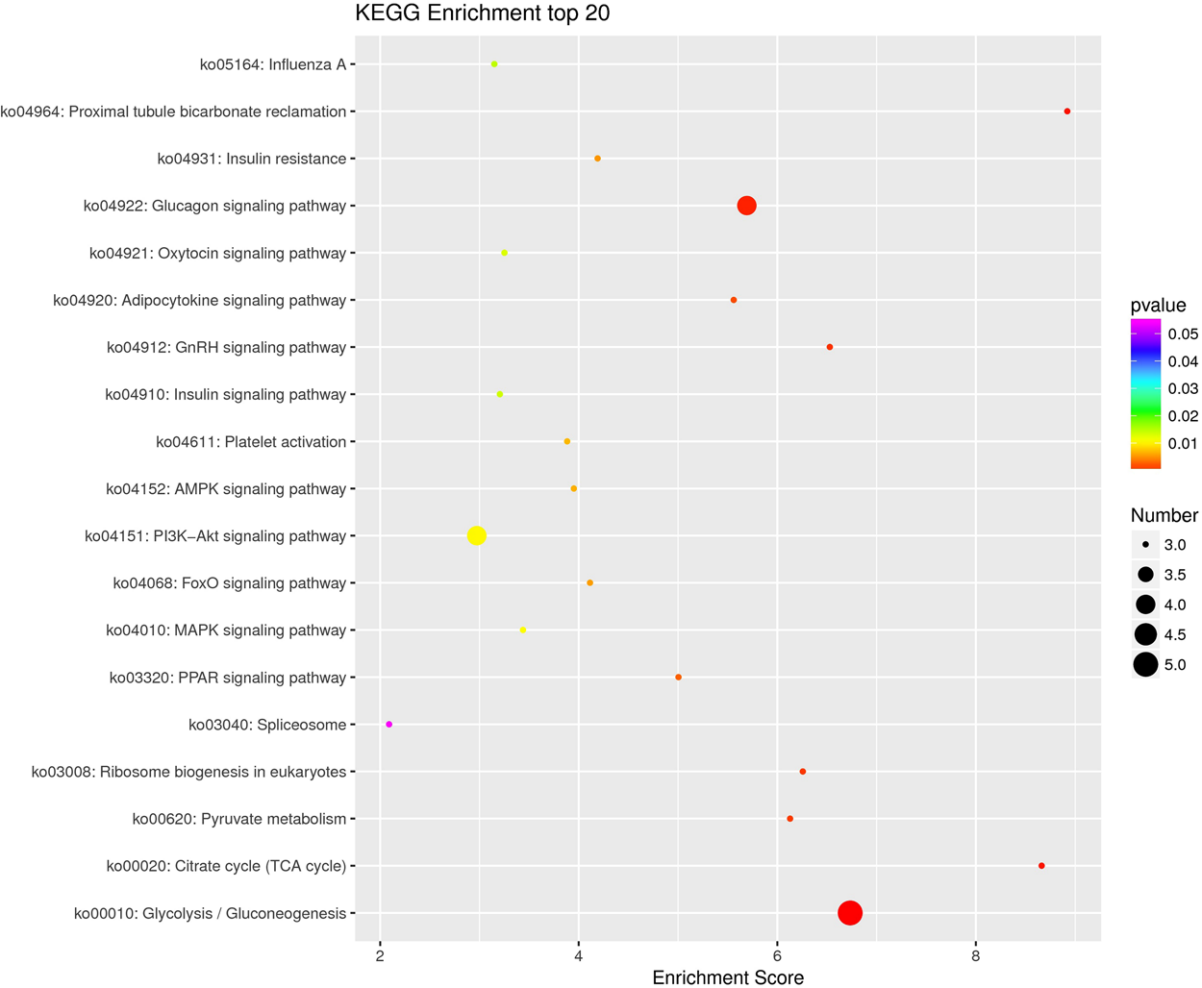
Top 20 significantly enriched KEGG pathways of putative target genes between testes and ovaries. KEGG pathways with *P* < 0.05 were considered significantly enriched. The color indicates a p-value from small (orange) to large (red). The smaller the p-value, the more vivid the red color. Each point corresponds to a pathway, and the larger the point is, the more genes are enriched in the pathway.

Furthermore, the predicted genes were submitted to KEGG analysis. The results showed that target genes were assigned into 141 signaling pathways. As shown in Fig. 4, glycolysis, gluconeogenesis and glucagon signaling pathways were the main metabolic pathways identified in this study (*P* < 0.05), indicating that these signaling pathways might play an important role in gonadal development. In addition, many predicted pathways were also involved in gonadal development, such as the MAPK pathway, GnRH signaling pathway, and insulin signaling pathway (*P* < 0.05). In addition to the pathways showed in Fig. 4, the Wnt signaling pathway, ovarian steroidogenesis, and estrogen signaling pathway were associated with gonadal development as well (Supplementary Table S2).

GO enrichment analysis different gonadal development stages (between ET and LT, EO and MO, MO and LO, respectively) showed that the main GO terms at the level of biological processes were “transcription, DNA-templated” and “regulation of transcription, DNA-templated”. At the level of cellular components, “cytoplasm” and “nucleus” were the main terms. At the molecular function level, “metal ion binding”, “ATP binding” and “zinc ion binding” were the dominant terms (Supplementary Fig. 3B–D). The KEGG results indicated that two signaling pathways “transcriptional misregulation in cancers” and “cGMP-PKG signaling pathway” were shared the most between the three groups (Supplementary Fig. 4A–C).

### Quantitative real-time PCR analysis of sex-biased miRNAs

**Figure 5:**
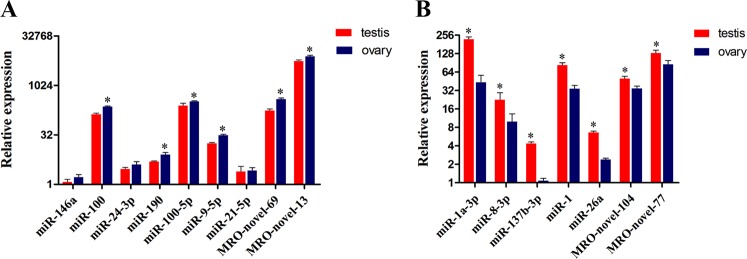
Expression patterns of sex-biased miRNAs confirmed by real-time PCR. The scales of the Y axis were indicated by Log2. The red column represent testis, while the blue column represents ovary. (**A**) Seven testis-biased miRNAs were highly expressed in the testis; (**B**) Nine ovary-biased miRNAs were highly expressed in the ovary. SnRNA was used as an internal reference. Bars represent the triplicate mean + SD from three individuals (n = 3). Asterisks indicate a significant difference (*P* < 0.05) between testis and ovary.

To validate the differential expression of sex-biased miRNAs identified by sequencing, we randomly selected five ovary-biased miRNAs (miR-146a, miR-100, miR-24-3p, miR-190, and MRO-novel-13) and three testis-biased miRNAs (miR-1a-3p, miR-8-3p, and miR-137b-3p) to analyze expression profiles using quantitative real-time PCR (qPCR). The qPCR results indicated that miR-146a, miR-100, miR-24-3p, miR-190, and MRO-novel-13 were up-regulated in ovary and miR-1a-3p, miR-8-3p, and miR-137b-3p were up-regulated in testis (Fig. 5), which was consistent with the sequencing results. Additionally, we selected some conservative and novel miRNAs to further characterize the DEMs between ovary and testis. MiR-100-5p, miR-9-5p, miR-21-5p, and MRO-novel-69 were highly expressed in ovary, whereas miR-1, miR-26a, MRO-novel-104, and MRO-novel-19 were highly expressed in testis. In general, the expression of DEMs was significantly different between the ovary and testis. For example, the expression level of miR-137-3p and miR-26a in the testis was four times and two times its concentration in the ovary, respectively. The resulting miRNA expression profiles were consistent with each other.

## Discussion

Although the importance of miRNAs has been recognized in regulating gene expression during sexual differentiation and gonadal development^7,8,25^, there is little information available regarding the expression of miRNAs in the prawn *M*. *rosenbergii*. Thus, the main objective of this study was to identify conserved and novel miRNAs present in *M*. *rosenbergii* and to evaluate differential expression patterns during gonadal development. Therefore, a series of DEMs and novel miRNAs were identified from seven miRNA libraries, and their corresponding target genes were predicted. Elucidation of the differences in miRNA and their sex-biased expression between tissues and stages will be valuable in understanding the gene expression regulatory networks underlying gonadal development.

The results of miRNA analysis revealed that U and A were the dominant nucleotides. This phenomenon may be related to some of the function of miRNA, such as binding to target genes^18^. Despite two peaks for the length distribution of small RNAs present in all libraries, the distribution is varied. As our results showed, the length distribution of small RNAs was roughly the same at various stages in gonads, but another differently peak was observed in MS and FS libraries (Fig. 2). In Decapod species *P*. *trituberculatus*, the amount of miRNAs and piRNAs (25–27 nt) was roughly the same^7^. However, piRNAs, with lengths ranging from 27 to 29 nucleotides, were the main enriched small RNA in ovarian tissue in *M*. *rosenbergii*. In contrast, miRNA was the dominant small RNAs observed in somatic tissues. This was also verified in *M*. *nipponense*^26^, suggesting that the length distribution and percentage of different small RNAs displayed specificity among various tissues as well as among species. Interestingly, after analysis of differently expressed piRNAs between ovary and testis, we found up regulation of 327 piRNAs in testis, while no piRNAs were unregulated in ovary (Supplementary Table S3). These results corroborate the previous study that piRNAs can affect the process of sperm production^27^.

In total, we obtained 1,954 known and 129 novel miRNAs from seven libraries. Among the 2,083 miRNAs, we focused on DEMs for the purpose of this study. From these we identified 17 ovary-biased and 24 testis-biased miRNAs in terms of expression patterns. These miRNAs could play a vital role in gonadal development. For instance, miR-194-5p and miR-148a-5p were more expressed in the ET library, suggesting possible involvement in early testicular development. In contrast, miR-24-3p was up-regulated in ovary, especially in the LO library, indicating that miR-24-3p could promote ovarian maturation. In addition, cgr-miR-122 was abundant in the ET library. Furthermore, GO enrichment analysis indicated that its target gene was MAPK 7, a key member in the MAPK signaling pathway, which regulates cyclin D1 expression during ovarian development and is therefore required for cell cycle progression^28^. Additionally, the testis-biased miRNA miR-193, target F-box/WD repeat-containing protein 7 (FBW 7) and could therefor suppress cell proliferation through the G2/M cell cycle transition^29^. Another testis-biased miRNA miR-145 was predicted to target cytosolic phospholipase. Cytosolic phospholipase has been verified to function in the synthesis of prostanoid involved in ovarian development in *Penaeus monodon*^30^. In addition, prediction results showed that many target genes participate in gonadal development, such as cyclin L1, HSP, and zinc finger protein^31–33^. For instance, Hsp110 is required for progression of spermatogenesis, and is associated with germ cell apoptosis^32^. The early growth response (Egr) family of zinc finger-containing transcription factors includes 4 members^34^. The Egr4 transcript and protein were localized in the developing murine testis and displayed distinct intracellular localization patterns within specific cell populations of the testis^35^. Additionally, some putative genes were mapped to reproductive biological pathways, and the main pathways included glycolysis, gluconeogenesis and the glucagon signaling pathway. Previous studies have shown that inefficient glycolysis combined with an increase in amino acid catabolism could be incompatible with the need for a high level of metabolism to maintain the rate of cell division required to form a gonad and populate it with germ cells^8^. Furthermore, Glucagon-like peptide 1 could be a factor involved in control of the hypothalamic-pituitary-gonadal axis^36^. Other pathways, such as the MAPK signaling pathway^11,37^, Wnt signaling pathway^38,39^, GnRH signaling pathway^40^, insulin signaling pathway^41^, cAMP signaling pathway^42^, ovarian steroidogenesis^43^, and the estrogen signaling pathway^44^ were proven to have influence on gonadal development. It is known that GnRH is the main hypothalamic regulator of the reproductive system in mammals^45^. Similar to results from previous studies on GnRH signaling pathway, lysophospholipase, 1-phosphatidylinositol 4,5-bisphosphate phosphodiesterase classes I and II-like, and MAPK 7 were mapped to the GnRH signaling pathway with p-value of approximately 0.01 in this study. Therefore, these DEMs and target genes are potentially involved in gonadal development. Further research is needed to confirm their regulatory mechanism mediated by miRNAs.

In conclusion, we provided the first report of known and novel miRNAs for *M*. *rosenbergii* gonad at different developmental stages. Based on the DEMs analysis, we identified 24 male-biased miRNAs and 17 female-biased miRNAs. These miRNAs may play an important role in gonadal development and reproduction. The GO and KEGG analysis of DEMs further revealed that some DEMs involved the process of gonadal development. The data obtained from our study could serve as a basis for further genetic mechanism research and contribute to valuable information for reproduction-control technology on *M*. *rosenbergii*.

## Materials and Methods

### Animals and sample collection

Healthy prawns were collected from a local fish market and transported back to our laboratory. The prawns were maintained at 26 ± 2 °C in a 30-L aerated aquarium for three days. Various samples, including ovaries, testes, and somatic tissue of female and male prawns were collected and immediately frozen in liquid nitrogen followed by storage at −80 °C. The developmental stages of the ovary and testis were determined according to the gonadal histological sections (Supplementary Fig. S5). Based on previous studies, ovarian development was divided into early, middle and late stages^40,46^, and testicular development was divided into early-middle and late stages^47^. Thus, seven samples including two samples of female and male somata, were prepared for the following RNA-Seq and qPCR experiments.

### RNA isolation

Total RNA was extracted using Trizol reagent (Invitrogen, USA) and treated with RNase-free DNase I (TaKaRa, Japan) for removing genomic DNA according to the manufacturer’s instructions. The extracted RNA was run on 1.5% agarose gel and Bioanalyzer 2100 system (Agilent Technologies, USA) for checking RNA integrity. The RNA quantity and purity were analyzed by a Nanodrop2000c spectrophotometer to obtain the A_260/280_ and A_260/230_ ratios with values within 1.90–2.10 and 2.00–2.50, respectively.

### Library construction and high-throughput sequencing

The libraries were constructed using NEB Next Multiplex Small RNA Library Prep Set for Illumina (New England Biolabs, USA) following the manufacturer’s instructions. Small RNAs were first isolated from the total RNAs of each of the seven samples. Then, these small RNAs were ligated to an activated 3′ and 5′ adaptor in turn. Then, adaptor-ligated small RNAs were reversed to create cDNA constructs. Subsequently, the generated cDNA constructs were amplified by PCR using Long AmpTaq 2 × Master Mix and SR Primer for Illumina. PCR products were purified on an 8% polyacrylamide gel. Finally, the seven resulting libraries were sequenced at OE Biotech. Co., Ltd. (Shanghai, China) using Illumina Solexa technology.

### Sequence data analysis

The initial sequence data from Illumina Solexa sequencing were filtered to remove low quality reads and adaptor sequences. Sequences originating from protein-coding genes (PCGs) were also removed by blast against the reference sequences derived from multi-transcriptomic datasets of *M*. *rosenbergii* in GenBank (SRX760286, SRX760284, SRX859032, SRX097639, SRX097638, and SRX092198) to obtain the final clean reads. The distribution of small RNA read lengths was determined. Then the reserved reads were classified into ribosomal RNA (rRNA), small nuclear (snRNA), and transfer RNA (tRNA) by blasting the Rfam database (version 10.0, http://rfam.xfam.org/). As there was no whole-genome data available for *M*. *rosenbergii*, the remaining sequences were searched against the miRBase database (version 21.0, http://www.mirbase.org/) to identify conserved miRNAs. Meanwhile, potential novel miRNAs were analyzed by Mirdeep2 and RNAfold^48^. The sequences exceeding a length of 18 bp were submitted to Mirdeep2 and the mapping genome sequences (*P* < 0.05) remained for further analysis. The remained sequences were able to predict its secondary structure, to be considered a novel miRNA. Subsequently, base-bias was analyzed for all miRNAs. To identify miRNAs with significantly different expression between samples, miRNA expression levels were normalized as transcripts per million (TPM): Normalized expression = (mapped read count / total reads) × 1.0 × 10^6^. Differentially expressed miRNAs (DEMs) analysis among libraries was carried out using Audic-Claverie^49^. The criteria for significant expression was defined as *P* < 0.05 and TPM > 2. The box-whisker plot allowed rough visualization of whether the data has symmetry and the degree of dispersion by using five statistics in the data: minimum value, the first quartile (25%), median (50%), third quartile (75%) and maximum value.

### Target gene prediction and functional annotation of DEMs

The raw sequences from transcriptomes of *M*. *rosenbergii* were trimmed and the overlapping high-quality reads were assembled to create longer contigs. Protein coding regions, 5′UTR, and 3′UTR were determined using ORF Finder (https://www.ncbi.nlm.nih.gov/orffinder/). Then the 3′UTR extracted from the above transcriptomes of *M*. *rosenbergii* were employed to predict potential target genes of the DEMs with Miranda software (S ≥ 150, ΔG ≤ −30 kcal/mol)^50^. All putative target genes were categorized into functional classes using Gene Ontology (GO) enrichment analysis (http://www.geneontology.org/), in which gene numbers were calculated for each term, and then hyper geometric testing was used to identify significantly enriched GO terms and the putative targets. We used Benjamini & Hochberg for the corrected p-value. GO terms with corrected values of *P* < 0.05 were defined as significantly enriched. Subsequently, pathway analysis of predicted target mRNAs was performed using the Kyoto Encyclopedia of Genes and Genomes (KEGG) database (http://www.genome.jp/kegg/pathway.html). KEGG pathways with *P* < 0.05 were considered significantly enriched.

### Quantitative real-time PCR

To verify the reliability of sequencing results, qPCR was employed on the sixteen randomly selected miRNAs. Total RNA (500 ng) was reverse transcribed using miRcute reverse transcriptase kit (Tiangen, China) according to the manufacturer’s instructions. Each reverse transcribed reaction mixture (20 μL) contained 5 μL of RNA (100 ng/μL), 2 × miRNA RT Reaction Buffer, 2 μL miRNA RT Enzyme Mix, and 3 μL H2O, with cycle parameters were 42 °C for 60 s and 95 °C for 3 min. Each miRNA was amplified by specific forward primers (Table 3) and a universal reverse primer (Tiangen, China) for qPCR. The qPCR was conducted using a Bio-Rad CFX96 Real-Time PCR Detection System (Bio-Rad). Each PCR reaction mixture (20 μL) contained 10 μL of 2 × miRcute Plus miRNA Premix, 1 μL of cDNA, 0.4 μL of forward and reverse primers, and 8.2 μL ddH2O. The PCR cycle parameters as follow, 95 °C for 15 min, 40 cycles of 94 °C for 20 s and 60 °C for 34 s. The miRNA expression levels were calculated using the 2^−△△CT^ method. Each sample was run in triplicate. SnRNA was amplified as an internal control^51^. The statistical significance was measured with one-way ANOVA by SPSS (version 20.0) and the differences were considered to be significant if *P* < 0.05.

**Table 3:** The primer sequences used in qPCR.

miRNA	primer sequence
miR-146a	TGTGAGAACTGAATTCCATGGGT
miR-100	GGAACCCGTAGATCCGAACTTA
miR-24-3p	GGCTCAGTTCAGCAGGAACAG
miR-190	CGGGAGATATGTTTGATATTCTTGG
miR-100-5p	GAACCCGTAGATCCGAACTTGT
miR-9-5p	TGGGTCTTTGGTTATCTAGCTGTATG
miR-21-5p	TGGGTAGCTTATCAGACTGATGTTG
miR-1a-3p	GGTGGGTGGAATGTAAAGAAGTATG
miR-8-3p	GGGTGGGTAATACTGTCAGGTAAAG
miR-137b-3p	GGGTTATTGCTTGAGAATACACGTAG
miR-1	GGGTGGAATGTAAAGAAGTATGGAG
miR-26a	TGGTTCAAGTAATCCAGGATAGGC
MRO-Novel-69	TGGGTCAAGTGTGGGACGT
MRO-Novel-13	TGATAGGTGGGAGGGTGGG
MRO-Novel-104	CGGTGGGTCTTTGGTGGTC
MRO-Novel-77	CGTGAGCAAAGTTTCAGGTGC
SnRNA	TTGGAACGATACAGAGAAGATTAGCAT

Note: The novel miRNAs have been named as MRO-novel-**, the others are known miRNAs. MRO, *Macrobrachium rosenbergii*.

## Supplementary information

Supplementary Information

Supplementary Table S1

Supplementary Table S2

Supplementary Table S3

## Data Availability

The datasets generated and analyzed during this are available from the corresponding author on reasonable request.
