# Supplementary material for: Identification and profiling of microRNAs during gonadal development in the giant freshwater prawn Macrobrachium rosenbergii
Source: Sci Rep. 2019 Feb 20;9:2406. doi: 10.1038/s41598-019-38648-x (PMC6382778; doi:10.1038/s41598-019-38648-x)
Supplement: Supplementary file 1 — Supplementary Information [file 41598_2019_38648_MOESM1_ESM.doc]

**Identification and profiling of microRNAs during gonadal development** **in the giant freshwater prawn *Macrobrachium rosenbergii***

Xue Liu^1,2,3,4^, Bi-Yun Luo^1,2,3,4^, Jian-Bin Feng^1,2,3,4^, Ling-Xia Zhou^1,2,3,4^, Ke-Yi Ma^1,2,3,4^*, Gao-Feng Qiu^1,2,3,4^*

^1^National Demonstration Center for Experimental Fisheries Science Education (Shanghai Ocean University). ^2^Key Laboratory of Exploration and Utilization of Aquatic Genetic Resources (Shanghai Ocean University), Ministry of Education. ^3^Key Laboratory of Freshwater Aquatic Genetic Resources, Ministry of Agriculture (Shanghai Ocean University). ^4^Shanghai Engineering Research Center of Aquaculture (Shanghai Ocean University)

*Corresponding author: Ke-Yi Ma and Gao-Feng Qiu, College of Fisheries and Life Science, Shanghai Ocean University, 999 Hucheng Huan Road, Pudong New Area, Shanghai 201306, China

TEL: 86-21-61900436; FAX: 86-21-61900436; E-mail: [kyma@shou.edu.cn](mailto:kyma@shou.edu.cn) (Ma); [gfqiu@shou.edu.cn](mailto:gfqiu@shou.edu.cn) (Qiu)

**Supplementary Information**

**Supplementary Figure S1.** **Nucleotide bias at each position on size distribution of miRNA sequences.** ET, early-middle testis; LT, late testis; EO, early ovary; MO, middle ovary; LO, late ovary; MS, male somata; and FS, female somata.

**Supplementary Figure S2.** **First nucleotide bias on size distribution of miRNA sequences.** ET, early-middle testis; LT, late testis; EO, early ovary; MO, middle ovary; LO, late ovary; MBM, MS, male somata; and FS, female somata.

**Supplementary Figure S3.** **Top 10** **GO terms of unigenes targeted by** **DEMs from different developmental stages of gonads.** GO analysis was performed for three categories: biological process, molecular function, and cellular component. A, beween ovary and testis; B, between ET and LT; C, between EO and MO, and D, between MO and LO. ET, early-middle testis; LT, late testis; EO, early ovary; MO, middle ovary; LO, late ovary; MS, male somata; and FS, female somata.

**Supplementary Table S1. GO annotation of the genes which were involved to gonadal development.**

**Supplementary Table S2. KEGG pathways which were associated with gonadal development.**

**Supplementary Figure S4. Top 20 KEGG pathways enriched by putative target genes from gonads at different developmental stages (between ET and LT (A), EO and MO (B), and MO and LO (C)).** ET, early-middle testis; LT, late testis; EO, early ovary; MO, middle ovary; LO, late ovary; MS, male somata; and FS, female somata.

**Supplementary Table S3. Expression profiles of 327 piRNAs up-regulated in the testes.**

**Supplementary Figure S5. Histological photographs of the ovarian and testicular development. Tissue sections were stained with stained with hematoxylin and eosin.** (A) Early-middle testis (ET) is mainly consisted of spermatogonia and spermatocytes. And spermatocytes nuclei were strongly stained by hematoxylin; (B) Late testis (LT) is mainly consisted of spermatozoa and nuclei were stained by hematoxylin; (C) Early ovary (EO) is mainly consisted of previtellogenic oocyte and its cytoplasm with strong staining of hematoxylin; (D) Middle ovary (MO) is mainly consisted of vitellogeneic oocyte and ooplasm becomes acidophilic and is stained by eosin; (E) Late ovary (LO) is mainly consisted of mature oocyte and germinal vesicle break down occurs. SG, spermatogonia; SC, spermatocytes; SZ, spermatozoa; Pvt, previtellogenic oocyte; Vt, vitellogeneic oocyte; GVBD, oocyte with germinal vesicle break down. N, nucleus. The scale bars indicate 50 μm.

**Supplementary Figure S1**


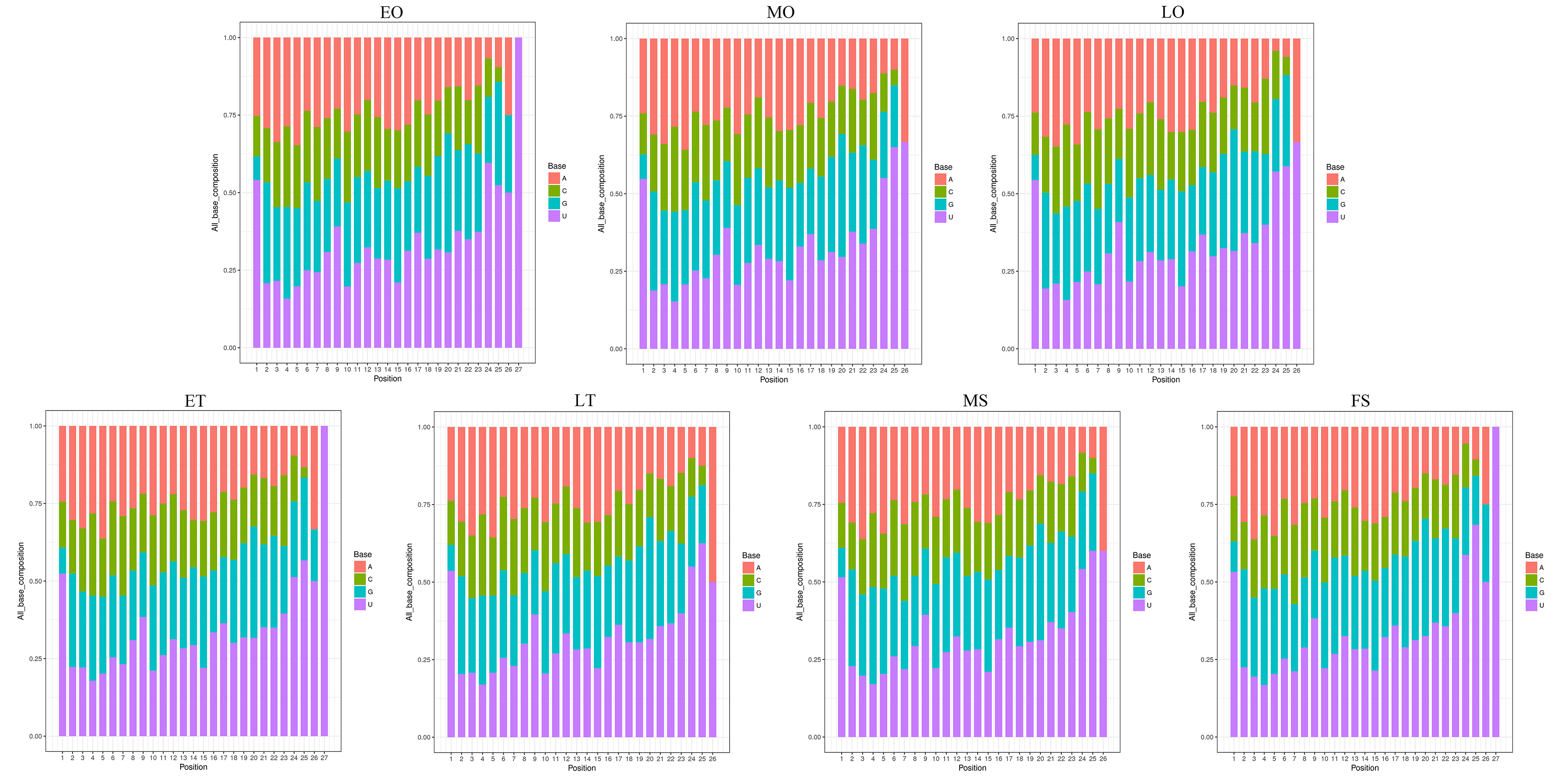


**Supplementary Figure S2**

**
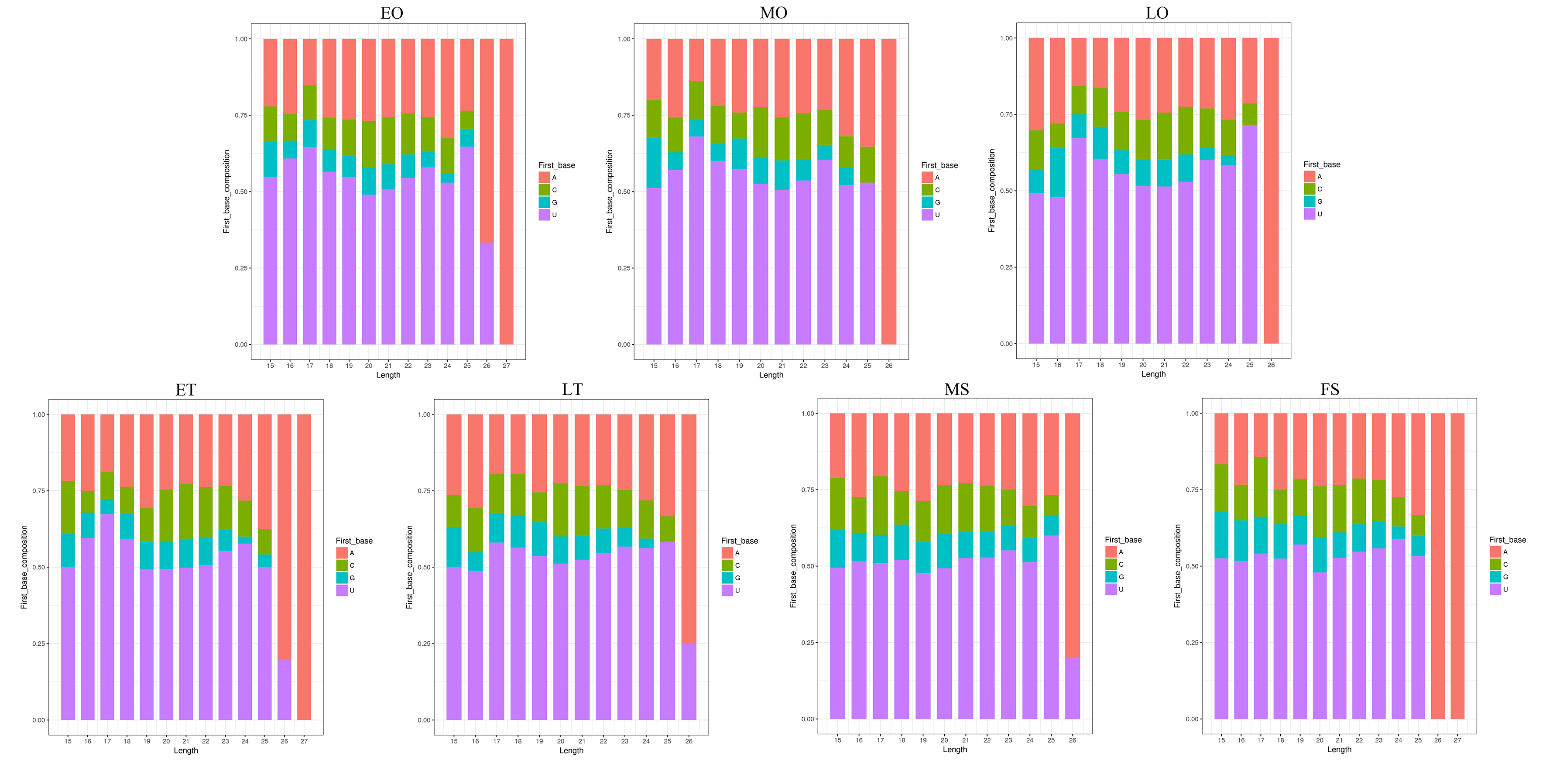
**

**Supplementary Figure S3**

**
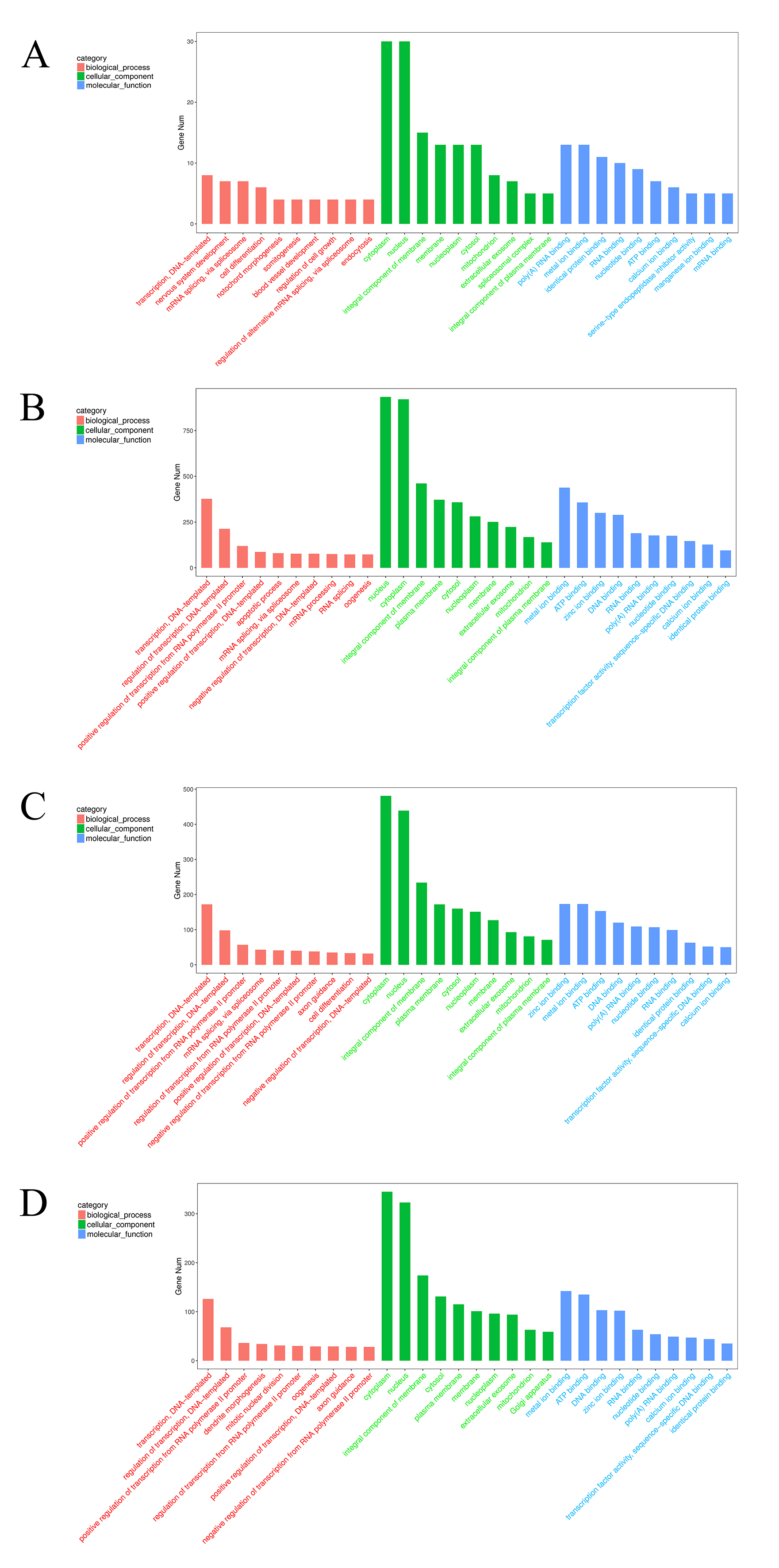
**

**Supplementary Figure S4**

**
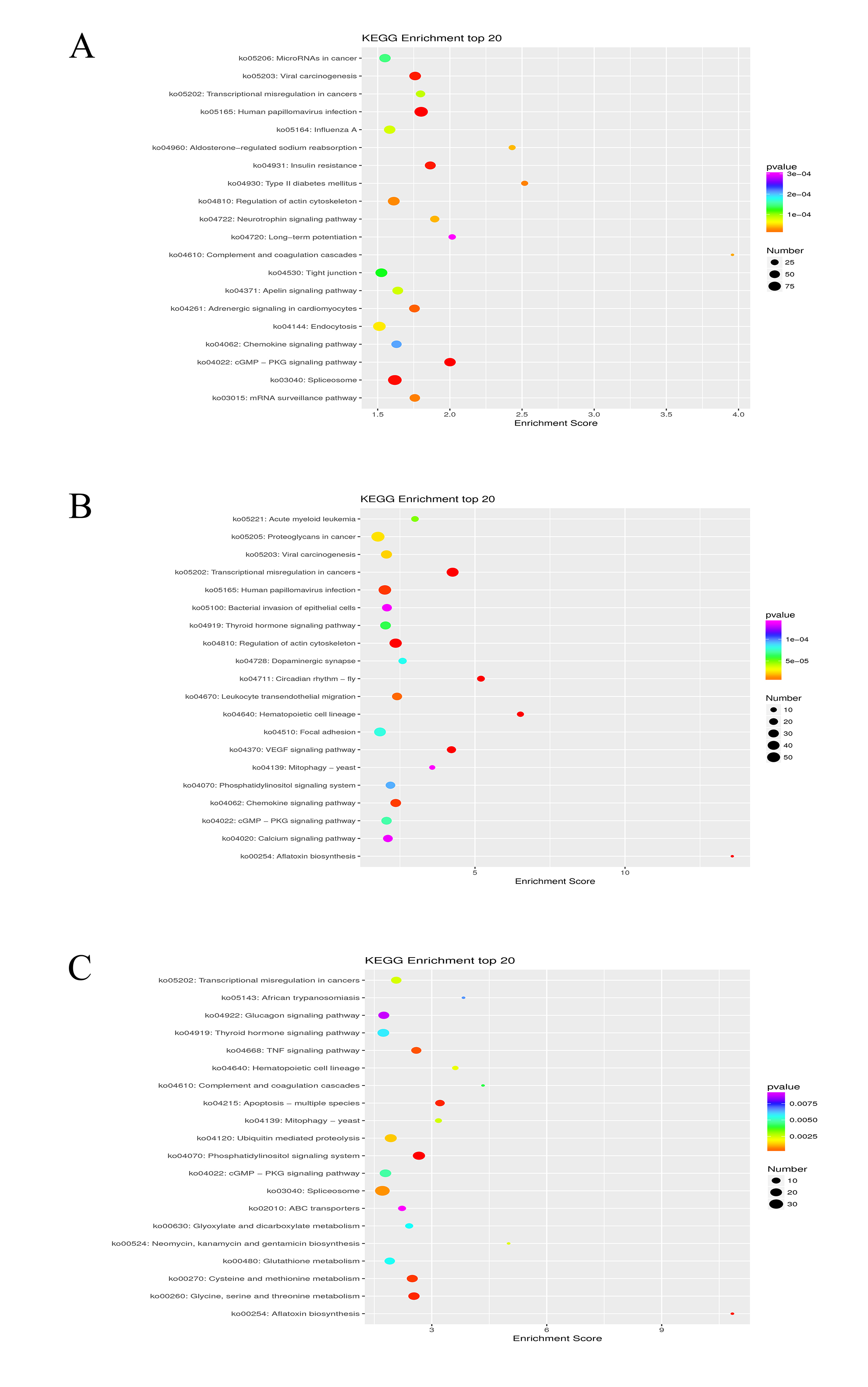
**

**Supplementary Figure S5**

**
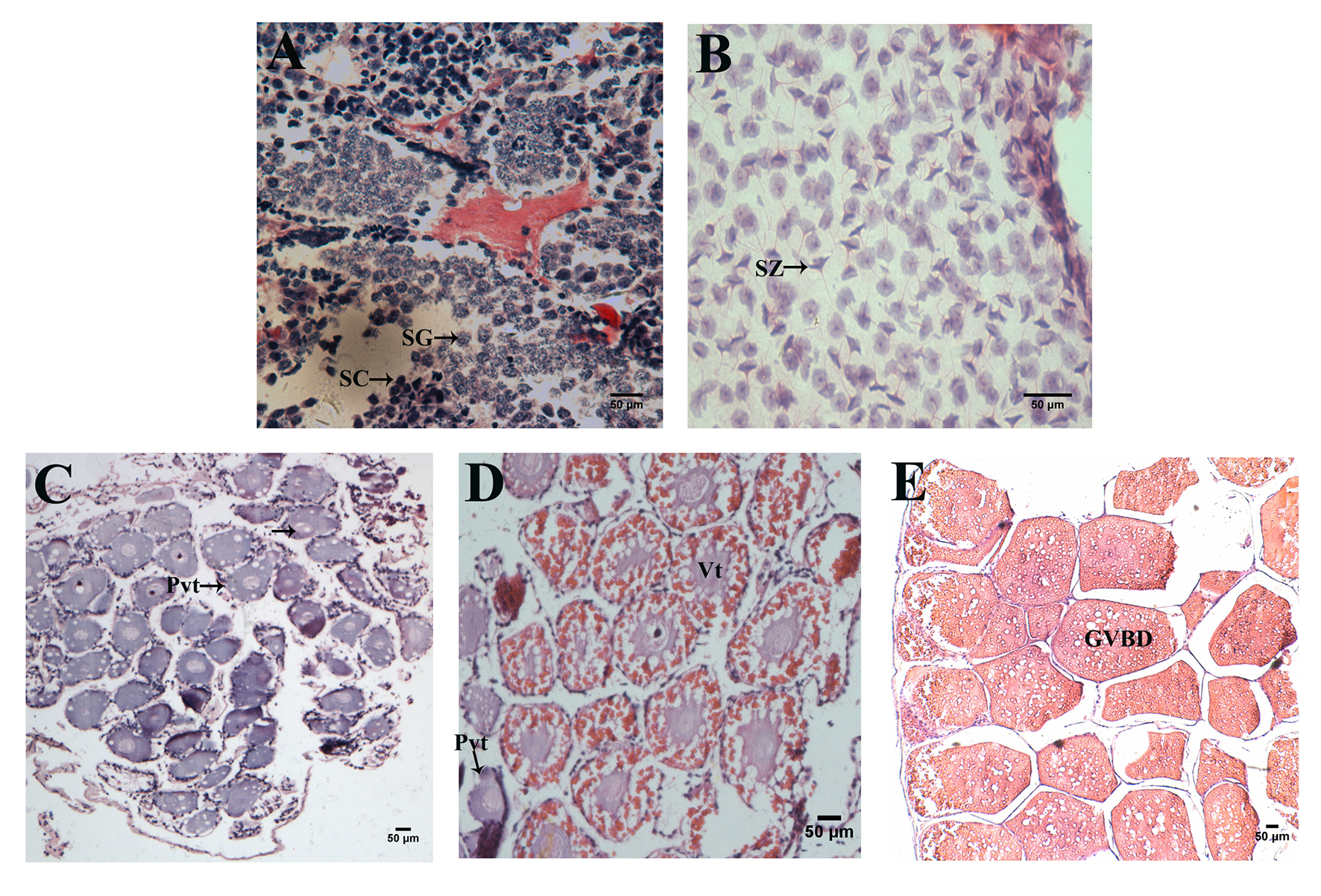
**
